# Supplementary figures and images for: Exploring snake occurrence records: Spatial biases and marginal gains from accessible social media
Source: PeerJ. 2019 Dec 17;7:e8059. doi: 10.7717/peerj.8059 (PMC6924322; doi:10.7717/peerj.8059)

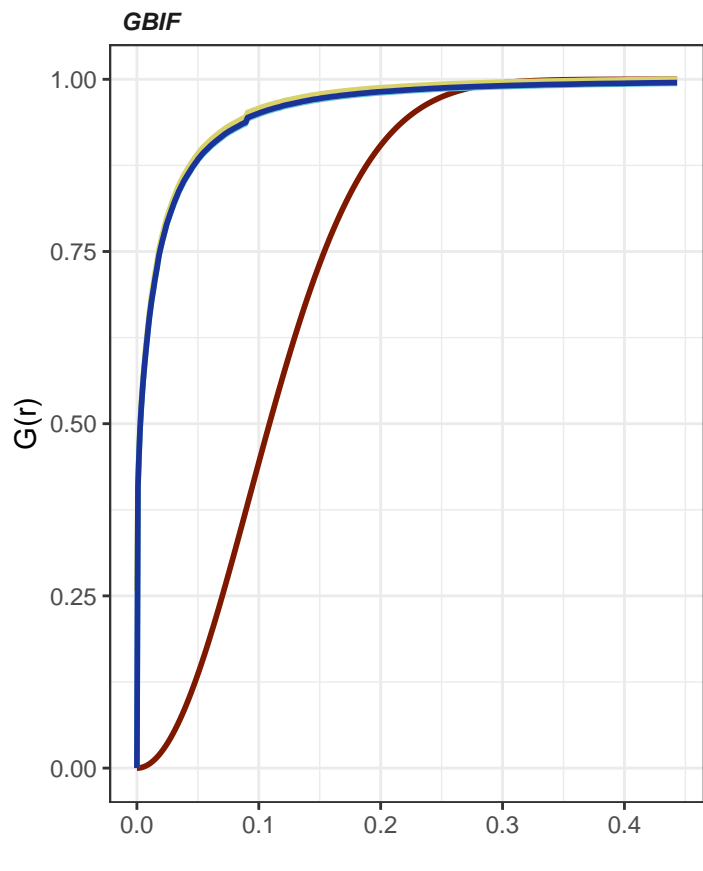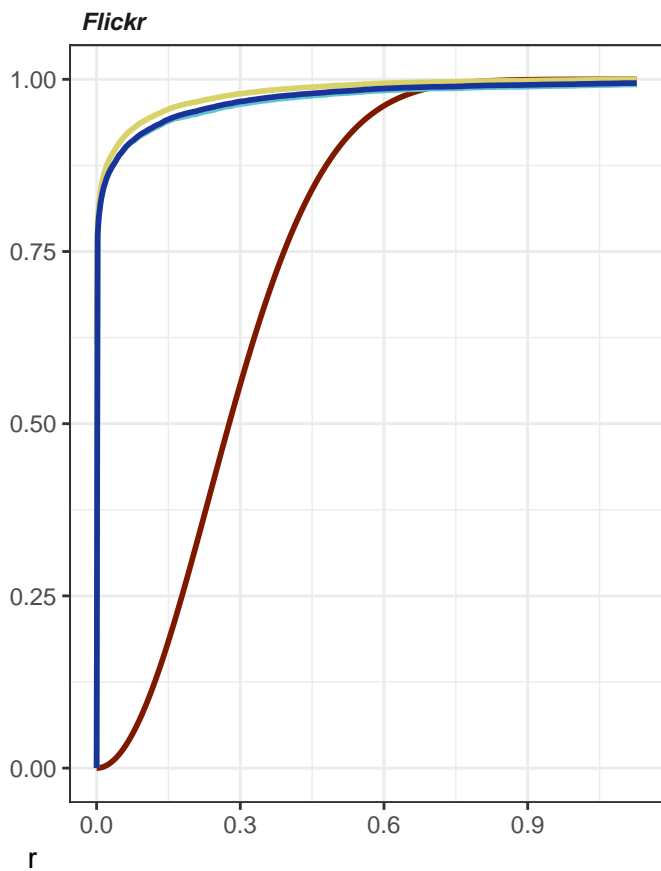

Supplement: Figure S1 [file peerj-07-8059-s002.pdf]

**GBIF**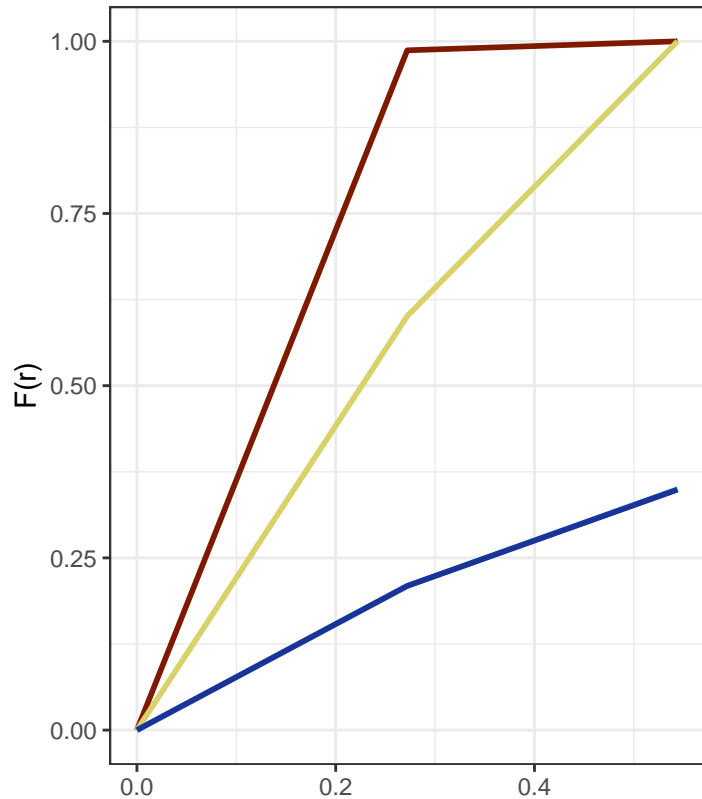**Flickr**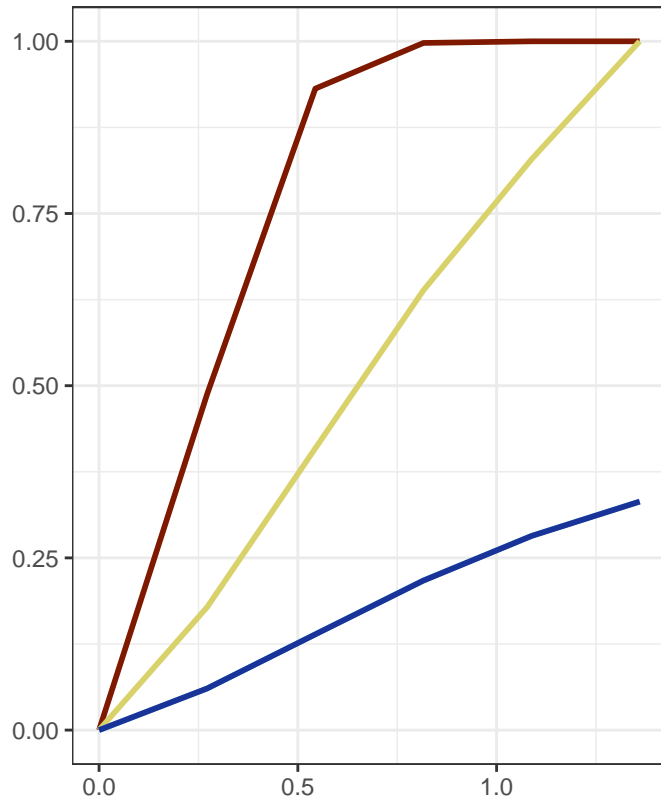

- $F_{pois}(r)$
- $F_{cs}(r)$
- $F_{bord}(r)$
- $F_{km}(r)$

Supplement: Figure S2 [file peerj-07-8059-s003.pdf]

**GBIF**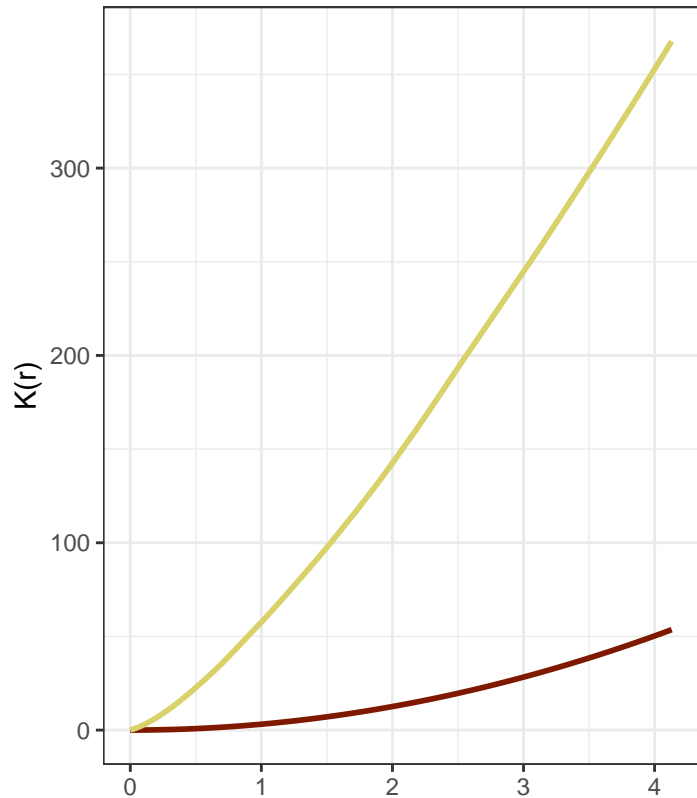**Flickr**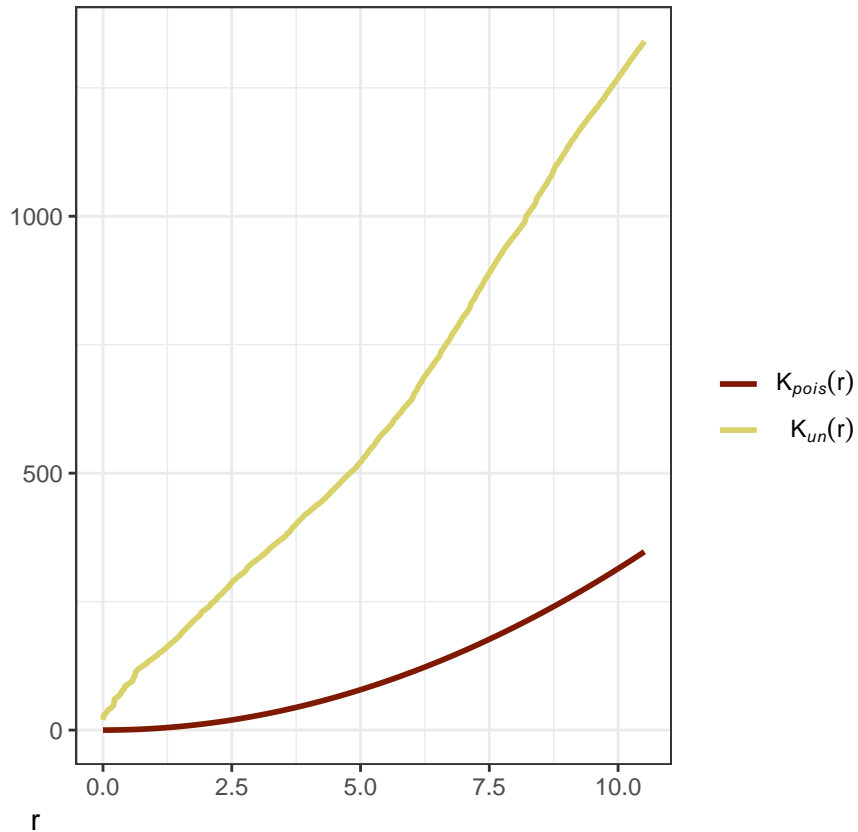

Supplement: Figure S3 [file peerj-07-8059-s004.pdf]

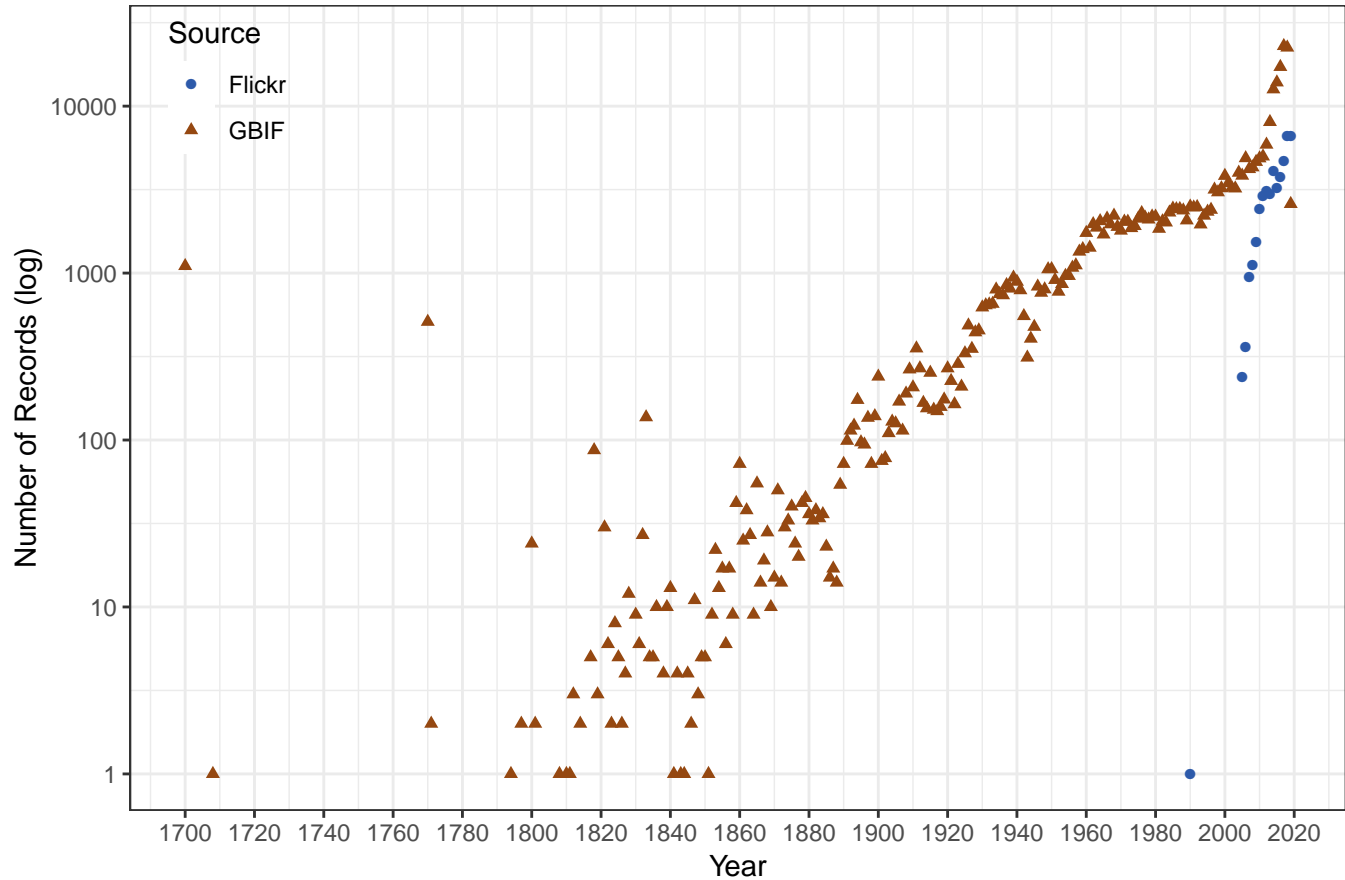

Supplement: Figure S4 — Red triangles show the per year number of GBIF records. Blue circles show the per year number of Flickr photographs, containing location data and tagged with the word snake. [file peerj-07-8059-s005.pdf]
